# Quality of Life Enhancement After Penetrating Keratoplasty in Keratoconus: A Vision-Related Functional Perspective

**DOI:** 10.3390/jcm14155325

**Published:** 2025-07-28

**Authors:** Anna Maria Gadamer, Piotr Miklaszewski, Dominika Janiszewska-Bil, Anita Lyssek-Boroń, Dariusz Dobrowolski, Edward Wylęgała, Beniamin Oskar Grabarek, Katarzyna Krysik

**Affiliations:** 1 Department of Ophthalmology, Trauma Centre, St. Barbara Hospital, 41-200 Sosnowiec, Poland; piotrmiklaszewski94@gmail.com (P.M.); dominika.bjaniszewska@gmail.com (D.J.-B.); anitaboron3@gmail.com (A.L.-B.); dardobmd@wp.pl (D.D.); kkrysik@gmail.com (K.K.); 2 Department of Ophthalmology, Faculty of Medicine, Academy of Silesia, 40-555 Katowice, Poland; 3 Collegium Medicum, WSB University, 41-300 Dabrowa Gornicza, Poland; bgrabarek7@gmail.com; 4 Department of Ophthalmology, District Railway Hospital, 40-760 Katowice, Poland; wylegala@gmail.com; 5 Department of Ophthalmology, Faculty of Medicine, Medical University of Silesia, 40-555 Katowice, Poland; 6 Faculty of Medicine and Health Sciences, Andrzej Frycz Modrzewski University, 30-705 Kraków, Poland

**Keywords:** quality of life, keratoconus, retrospective studies, visual acuity, penetrating keratoplasty

## Abstract

**Background/Objectives**: Keratoconus (KC) is a bilateral asymmetric corneal ectasia characterized by progressive corneal thinning, irregular astigmatism, and impaired visual acuity. The National Eye Institute (NEI) developed the Visual Function Questionnaire (VFQ-25) to assess the impact of visual impairment on quality of life. This study aimed to evaluate the effect of penetrating keratoplasty (PKP) on quality of life and visual acuity in KC patients one year postoperatively. **Methods**: A retrospective study was conducted between January 2018 and December 2022 at the Ophthalmology Department of Saint Barbara Hospital, Trauma Center, Sosnowiec, Poland. A total of 71 patients (86 eyes) diagnosed with KC underwent PKP. The VFQ-25 questionnaire and visual acuity measurements were assessed preoperatively and one year postoperatively. **Results**: The study cohort included 71 patients (20 females, 28.17%; 51 males, 71.83%). Preoperative visual acuity ranged from less than 0.05 on the Snellen chart to 0.5. Postoperatively, visual acuity improved to a range of 0.1–1.0. A visual acuity of 1.0 was achieved in 21 eyes (24.42%; 5 females, 24%; 16 males, 76%), with a statistically significant improvement (*p* < 0.01). The mean VFQ-25 composite score increased from 57.96 (±17.58) preoperatively to 81.42 (±14.66) postoperatively (*p* < 0.001). Domains with the lowest preoperative scores were “role difficulties,” “general vision,” and “mental health,” while “color vision” scored highest. **Conclusions**: PKP significantly enhances both objective visual acuity and subjective quality of life in KC patients, as reflected in VFQ-25 questionnaire outcomes.

## 1. Introduction

Keratoconus (KC) is a progressive, bilateral, non-inflammatory corneal ectasia characterized by asymmetric thinning and protrusion of the cornea, leading to irregular astigmatism and significant visual impairment [1,2,3,4]. Typically manifesting during adolescence or early adulthood, the disease often progresses over several decades, causing worsening visual acuity, distortion, and increased sensitivity to light [5]. These visual limitations can substantially interfere with everyday activities such as driving, reading, and recognizing faces and are frequently associated with reduced emotional well-being and mental health challenges [6,7,8].

The global prevalence of KC varies across populations, with reported estimates ranging from 1.38 to over 5 per 1000 individuals, depending on ethnicity, diagnostic criteria, and environmental factors [9,10,11,12]. Given its early onset and chronic progression, KC presents a growing public health concern, with increasing implications for healthcare systems and quality of life outcomes in affected individuals [13,14].

Management of KC depends on disease severity [15]. Early-stage cases are often managed conservatively with spectacles or rigid gas permeable contact lenses [15]. However, as the condition advances and the corneal structure becomes increasingly irregular, non-surgical options may no longer provide adequate visual correction [15]. In these cases, surgical interventions are indicated to stabilize the cornea and improve vision. Therapeutic options include corneal cross-linking, intracorneal ring segments, deep anterior lamellar keratoplasty (DALK), and penetrating keratoplasty (PKP) [16].

DALK is typically preferred in advanced keratoconus without corneal scarring, due to its lower risk of endothelial rejection and superior long-term graft survival. PKP, by contrast, is primarily reserved for cases with central corneal scarring—such as those following acute hydrops—or where lamellar procedures are not feasible [17,18,19,20].

As KC affects not only objective vision but also subjective functioning and quality of life, understanding patients’ lived experiences is critical to guiding treatment decisions. The National Eye Institute (NEI) developed the Visual Function Questionnaire (VFQ-25), which is one of the most widely used instruments for assessing vision-related quality of life [17,18,19,20]. It evaluates multiple functional domains, including near and distance vision, mental health, role difficulties, and social functioning. The VFQ-25 has been validated in numerous ocular diseases, including keratoconus, glaucoma, and cataract [21,22]. Kandel et al. [23] systematically reviewed the use of vision-related quality of life questionnaires in keratoconus and identified 18 different tools across 45 studies. While most instruments were general ophthalmic surveys not specific to KC, NEI VFQ-25 emerged as the most frequently applied measure. Their review emphasized that surgical and non-surgical interventions for KC—ranging from spectacles and contact lenses to corneal cross-linking and keratoplasty—can lead to substantial improvements in visual functioning and patient-perceived outcomes.

Although numerous studies have reported anatomical and visual improvements following PKP, relatively few have examined its long-term impact on patient-reported quality of life using validated instruments [24,25,26]. Moreover, the literature remains inconsistent in correlating functional outcomes such as best-corrected visual acuity and contrast sensitivity with subjective improvements in daily life [20,27,28]. Therefore, a more integrated evaluation that combines both clinical and patient-reported metrics is needed to guide evidence-based treatment and enhance patient counseling [20,27,28].

Therefore, this study aimed to evaluate the impact of penetrating keratoplasty on both visual acuity and vision-related quality of life in patients with keratoconus. The NEI VFQ-25 questionnaire was used as the primary outcome measure, assessed before and one year after surgery. By correlating subjective and objective parameters, this study seeks to provide new insights into the functional effectiveness of PKP and its implications for clinical decision-making in advanced KC.

## 2. Materials and Methods

### 2.1. Patients

The study was conducted between January 2018 and December 2022 at the Ophthalmology Department of Saint Barbara Hospital, Trauma Center, Sosnowiec, Poland. The choice of this facility ensured that all participants underwent standardized ophthalmic assessments and received consistent follow-up care, thereby enhancing the reliability and validity of the collected data. All procedures were conducted in accordance with institutional guidelines to ensure patient confidentiality and data protection. This section was developed based on our previously published work [29]. The inclusion and exclusion criteria are presented in Table 1.

### 2.2. Study Design

This retrospective study was based on clinical and questionnaire data that had been prospectively collected as part of a quality improvement protocol for keratoconus patients undergoing PKP. VFQ-25 questionnaires and visual acuity assessments were conducted preoperatively and one year postoperatively and later retrospectively analyzed for this research.

This study aimed to evaluate the impact of PKP on both objective visual function and subjective quality of life in patients with KC. A particular focus was placed on assessing changes in vision-related quality of life using the VFQ-25, which served as the primary outcome measure of this study.

To achieve this, all participants underwent comprehensive ophthalmic evaluations both before PKP and one year postoperatively. These assessments included best-corrected visual acuity (BCVA) measurements using the Snellen chart; contrast sensitivity was assessed using the Pelli–Robson chart to evaluate the ability to distinguish objects at low contrast levels, intraocular pressure measurements with an i-care tonometer, and slit-lamp biomicroscopy. Preoperative BCVA was measured using RGP contact lenses, which are standard for achieving optimal correction in patients with keratoconus. Fundus examinations were performed under pupillary dilation with 1% Tropicamide to rule out posterior segment pathology. Postoperative BCVA was assessed using a Snellen chart at a distance of 6 m under standardized photopic conditions, with best optical correction determined by full subjective refraction using trial lenses.

Additionally, contrast sensitivity testing was included in the assessment of visual function, as it provides crucial insights into functional vision beyond standard visual acuity measurements. Evaluating contrast sensitivity is particularly relevant for KC patients, as the disease affects not only sharpness of vision but also visual quality under different lighting conditions.

Preoperative corneal parameters, including anterior keratometry, astigmatism, and average keratometry, were assessed using the Pentacam HR and Swept-Source Optical Coherence Tomography (Casia SS-1000, Tomey, Nagoya, Japan). The comparison of corneal measurements between these two devices aimed to assess their consistency and reliability in measuring corneal curvature and thickness, which is essential for ensuring accurate postoperative monitoring. These data were used descriptively to support the clinical indication for surgery.

The VFQ-25 questionnaire, the primary tool used in this study, was administered preoperatively and at the one-year follow-up to evaluate changes in vision-related quality of life. The questionnaire assessed various domains, including general vision, near and distance activities, social functioning, mental health, and role difficulties.

To complement patient-reported outcomes, all participants underwent comprehensive preoperative and postoperative visual function testing. BCVA was measured using a Snellen chart under standardized conditions. Contrast sensitivity was assessed with the Pelli–Robson chart, providing information on low-contrast visual performance, which is particularly relevant in keratoconus due to its impact on irregular astigmatism and higher-order aberrations. Preoperative corneal morphology was assessed using Pentacam HR (Scheimpflug imaging) and Swept-Source Optical Coherence Tomography (SS-OCT; Casia SS-1000), offering high-resolution corneal curvature, pachymetry, and anterior segment profiles. These objective metrics supported surgical decision-making. However, due to the retrospective nature of the study and variable follow-up imaging protocols, postoperative Pentacam and OCT data were not consistently available for all participants and were therefore not included in the statistical analysis.

By integrating objective visual assessments, corneal imaging, and patient-reported outcomes, this study provided a comprehensive evaluation of the effectiveness of PKP in restoring both functional vision and quality of life for patients with advanced KC.

### 2.3. Visual Function Questionnaire

Patients participated in the assessment using the NEI VFQ-25, version 2000, which was specifically translated in [30,31] to ensure that language barriers did not influence the accuracy of responses. In most cases, participants completed the questionnaire independently. However, for patients with impaired visual acuity that hindered them from reading the questions, the questionnaire was administered by the attending physician in the form of a structured medical interview. The questionnaire responses, along with visual acuity measurements, were collected both before the PKP procedure and at the one-year follow-up.

The NEI VFQ-25 questionnaire (version 2000, Interviewer-Administered Format) was used to evaluate vision-related quality of life. It contains 25 core items and optional supplementary questions grouped into 12 subscales: general health, general vision, ocular pain, near vision, distance vision, vision-specific social functioning, vision-specific mental health, vision-specific role difficulties, vision-specific dependency, driving, color vision, and peripheral vision. Each subscale addresses a distinct aspect of visual function or its impact on daily life and emotional well-being.

The questions explore various levels of visual performance, including difficulty with reading, driving, recognizing faces, or navigating stairs, as well as psychological effects such as frustration or fear of embarrassment due to poor vision. A detailed description of the type of questions included in each subscale is presented in Table 2.

Scoring was performed according to NEI guidelines, with responses transformed to a 0–100 scale, where higher scores indicate better visual functioning.

### 2.4. Scheimpflug Imaging System (Pentacam HR)

The Pentacam HR (Oculus, Wetzlar, Germany) employs Scheimpflug imaging to generate high-resolution, three-dimensional corneal topography. It provides essential parameters such as corneal curvature, pachymetry, and anterior chamber depth, facilitating the evaluation of corneal integrity following PKP. The system’s automated alignment function ensures reproducible and reliable measurements [32,33,34].

### 2.5. Swept-Source Optical Coherence Tomography (OCT Casia)

The OCT Casia (Tomey, Nagoya, Japan) is an advanced imaging device that provides high-resolution cross-sectional images of the cornea. Using a 1310 nm swept-source laser, it enables precise measurements of both anterior and posterior corneal surfaces, offering detailed corneal curvature and thickness data. This technology is instrumental in assessing structural changes pre- and PKP [32,33,35].

### 2.6. Statistical Analysis

All analyses were performed using the R statistical computing environment, version 4.4.0. Continuous data and survey results were primarily presented as medians with interquartile ranges (Q1–Q3), along with minimum and maximum values, due to deviations from normal distribution verified by the Shapiro–Wilk test.

For the VFQ-25 composite score, mean ± standard deviation was also reported to enable comparison with previously published studies, although all statistical comparisons were performed using non-parametric methods. Differences between pre- and postoperative survey responses and device measurements were calculated, including the percentage absolute difference, defined as the absolute value of the difference between two time points or devices divided by the maximum of the two values, multiplied by 100 to express the change as a percentage.

Importantly, VFQ-25 scores were analyzed at the patient level, as each participant completed one questionnaire before and one year after surgery. In cases of bilateral PKP, only one VFQ-25 score per patient was included to avoid duplication and intra-cluster correlation. In contrast, eye-level data, such as best-corrected visual acuity (BCVA), contrast sensitivity, and corneal imaging parameters, were analyzed separately for each eye.

The Wilcoxon signed-rank test was used to compare pre- and postoperative values. Median differences are presented in the tables. Additionally, the percentage distribution of changes (“+”, “0”, “−”) was provided to indicate the direction of change after PKP (increase, no change, decrease). A two-sided *p*-value of <0.05 was considered statistically significant.

To explore the relationship between subjective quality of life and objective visual function parameters, Spearman’s rank correlation analysis was conducted. Specifically, correlations were assessed between the preoperative VFQ-25 composite scores and the following preoperative parameters: BCVA, contrast sensitivity, and average keratometry (Kmean) obtained from Pentacam HR. Spearman’s ρ coefficients and corresponding *p*-values were calculated to evaluate the strength and significance of these associations.

#### Sample Size Analysis

A power analysis was performed to estimate the minimum required sample size for detecting statistically significant differences in VFQ-25 scores and visual acuity before and after penetrating keratoplasty (PKP). An effect size (Cohen’s d) of 0.8 was assumed, representing a large effect. This decision was based on prior literature that demonstrated substantial improvements in quality of life and functional vision outcomes among keratoconus (KC) patients undergoing PKP. Using G*Power 3.1 software [36], with a significance level (α) of 0.05 and a power of 0.80, the minimum required sample size for paired-sample comparisons was calculated to be 52 patients. The actual sample included 71 patients (86 eyes), which exceeded this requirement and ensured adequate statistical power. A post hoc power analysis further confirmed that the study retained sufficient power (>0.85) to detect changes in VFQ-25 scores, reinforcing the robustness and reliability of the findings.

## 3. Results

### 3.1. Demographic and Clinical Characteristics of the Study Group

A total of 71 patients (86 eyes) with advanced keratoconus underwent penetrating keratoplasty (PKP) and were included in the analysis. The cohort comprised 20 females (28.17%) and 51 males (71.83%). The mean age at the time of surgery was 36.80 ± 9.66 years (38.11 ± 11.16 in females; 34.93 ± 9.25 in males).

### 3.2. VFQ-25 Version 2000 Outcomes

A significant improvement in vision-related quality of life was observed following PKP. The VFQ-25 composite score increased from a preoperative mean of 57.96 ± 17.58 to 81.42 ± 14.66 at the one-year follow-up (*p* < 0.001, Wilcoxon signed-rank test). Statistically significant improvements were documented across all 12 subscales (*p* < 0.001 for each), with the most pronounced median increases occurring in the domains of near activities, distance activities, and general vision. Preoperatively, the lowest scores were recorded in role difficulties, general vision, and mental health, underscoring the psychosocial and functional impact of keratoconus. In contrast, the color vision domain demonstrated the highest baseline values and minimal postoperative change, consistent with the limited influence of keratoconus on chromatic discrimination. A detailed summary of all subscale outcomes, including median values, changes, and responder distributions, is provided in Table 3.

Improvements in contrast sensitivity and BCVA paralleled the significant gains observed in VFQ-25 subscales, especially in near activities, distance vision, and general vision, supporting the functional recovery perceived by patients. While a formal statistical correlation between subjective and objective outcomes was beyond the scope of this analysis, the observed trends align with expected structure–function relationships. Objective parameters provided clinically relevant confirmation of the VFQ-25 outcomes.

### 3.3. Visual Acuity and Contrast Sensitivity

Objective measures of visual function showed marked improvement after surgery. Median BCVA improved from 0.05 [0.01; 0.20] preoperatively to 0.80 [0.60; 1.00] postoperatively (*p* < 0.001), as presented in Table 4. Similarly, contrast sensitivity, measured using the Pelli–Robson chart, increased from 1.10 [0.90; 1.35] to 1.65 [1.50; 1.80] (*p* < 0.001), as shown in Table 5.

### 3.4. Preoperative Corneal Imaging (Pentacam HR and OCT Casia)

Preoperative keratometric values were assessed using both Pentacam HR and Casia SS-1000 to evaluate baseline corneal structure and ensure measurement reliability. The two imaging systems demonstrated strong agreement in anterior and posterior keratometry as well as astigmatism values, confirming the consistency of structural assessment between devices. These results, while not central to the primary outcomes, provide context for the preoperative status of the cornea. Detailed comparative data between devices were published in a previous study [29]. The current analysis does not include postoperative corneal imaging.

### 3.5. Correlation Between Subjective and Objective Outcomes

To explore the relationship between vision-related quality of life and objective clinical parameters, a Spearman correlation analysis was performed using preoperative data. A statistically significant negative correlation was found between VFQ-25 composite scores and BCVA (ρ = −0.48, *p* < 0.001), indicating that poorer visual acuity was associated with lower perceived quality of life. A significant positive correlation was also observed between VFQ-25 scores and contrast sensitivity (ρ = 0.41, *p* < 0.001). However, no significant correlation was identified between VFQ-25 scores and average keratometry (ρ = −0.12, *p* = 0.28). These findings suggest that quality of life in keratoconus patients is more closely aligned with functional visual outcomes than with corneal curvature measurements.

## 4. Discussion

In this study, we evaluated the quality of life in keratoconus (KC) patients before and one year after penetrating keratoplasty (PKP) using the NEI VFQ-25 questionnaire. Our findings clearly demonstrate that PKP significantly improves both visual acuity and vision-related quality of life.

The VFQ-25 was easy and quick to administer, resulting in a high response rate and minimal response bias due to fatigue or misunderstanding [37]. This aligns with previous studies that have validated the VFQ-25 as a reliable and practical tool for assessing patient-reported outcomes in KC and other corneal pathologies [13,38]. Its user-friendly structure supports accurate self-assessment and reduces the likelihood of incomplete or inconsistent responses [38,39,40].

Before PKP, the lowest scores were observed in the subscales of “role difficulties,” “general vision,” and “mental health,” consistent with findings by Mahdaviazad et al. [41], who reported low scores in “ocular pain,” “general vision,” and “mental health.” Similar psychological burdens have been observed in elderly patients with AMD, where poor vision is associated with fear of falling and diminished mental well-being [42]. These findings suggest that vision-related psychological distress occurs across age groups and ocular diseases.

In addition, a cross-sectional study from Egypt (Zagazig University Hospitals) administering the VFQ-25 to 54 keratoconus patients reported a mean composite score of 73.97 ± 15.11, with the lowest subscale scores in “general vision” (64.2 ± 13.3) and “ocular pain” (49.9 ± 13.7). QoL was strongly associated with demographic factors including age, education, employment, smoking status, and marital status (*p* < 0.001) [43].

Similarly, a nationwide study in Saudi Arabia (n = 429) reported mean VFQ-25 composite scores of 58.6 ± 18.0, with younger age and female sex associated with worse outcomes. Importantly, low vision aid usage significantly improved scores [20]. A hospital-based study in Nepal demonstrated a decline in VFQ-25 scores across KC severity grades, supporting the progressive impact of disease on visual function [44]. Compared with healthy controls, all subscale scores were significantly lower in KC patients, underscoring the profound effect of the disease on daily functioning. Chiraples et al. also noted particularly poor scores in “general vision” and “ocular pain” among patients undergoing “epi-on” cross-linking, highlighting similar challenges in visual tasks such as navigating stairs or reading signs in low-light conditions [38]. Interestingly, our cohort reported relatively high preoperative scores in “dependency” and “color vision,” suggesting maintained independence in familiar environments and preserved chromatic discrimination—consistent with reports in the literature indicating that KC primarily impairs spatial resolution, not color perception [13,20,38]. Our results align with Al Zabadi et al. [13], who found that KC patients had significantly reduced scores in domains such as general and near vision, mental health, and social functioning, and scores in “ocular pain” and “color vision” were relatively low [13].

These results are further supported by a systematic review conducted by Vreijsen et al. [45], which analyzed 14 longitudinal studies and reported improvements in both vision-related and general health-related QoL following corneal transplantation. The review emphasized the predictive value of preoperative visual function and postoperative BCVA for QoL outcomes and advocated for routine use of patient-reported outcome measures (PROMs) in patient-centered care [45].

In a recent systematic review and meta-analysis, Kijonka et al. [46] synthesized data from 21 studies encompassing 1539 eyes treated with PKP or DALK. The authors reported statistically significant improvements in both visual acuity and astigmatism after surgical intervention, using a random-effects model to account for study heterogeneity. Notably, the average improvement in visual acuity was −0.383 logMAR, and astigmatism was reduced by −0.357 diopters. These results align closely with our VFQ-25 outcomes, emphasizing the substantial functional gains that patients experience post-keratoplasty and reinforcing the importance of combining objective visual metrics with patient-reported outcomes [46].

Aydin Kurna et al. [47] reported that visual acuity in the better eye strongly correlates with QoL—especially in the “distance vision” and “mental health” domains—echoing our own findings. Likewise, Yildiz et al. [48] and Lim et al. [49] observed improvements in BCVA (to 20/40 or better) after PKP and significantly increased VFQ-25 scores, with Yildiz et al. reporting a rise from 55.2 ± 19.7 to 84.3 ± 6.6, comparable to our result (81.42 ± 14.66). Panthier et al. [38], similarly found that poor vision in the better eye significantly reduced vision-related QoL.

Other interventions for KC yield similar findings. Rodrigues et al. [50] reported improvements in BCVA and VFQ-25 after ICRS implantation, with a significant negative correlation between visual acuity and QoL (r = −0.40, *p* = 0.001) [50].

Niziol et al. [51] similarly demonstrated durable improvements with a mean VFQ-25 score of 84.5 ± 12.1 over two decades post-surgery, highlighting the lasting benefits of keratoplasty [51].

Our correlation analysis further confirmed that subjective QoL is more strongly influenced by visual function—especially BCVA and contrast sensitivity—than by anatomical severity (e.g., corneal curvature). This supports findings by Lee et al. [52], who demonstrated that BCVA in the better eye was the strongest predictor of total VFQ-25 scores across multiple ocular disorders.

Nevertheless, the benefits of PKP must be balanced against potential risks. In a retrospective study, Soleimani et al. [53] reported a 1.7% incidence of post-keratoplasty infectious keratitis, most commonly caused by Streptococcus viridans and Staphylococcus aureus. Notably, infection rates did not significantly differ between PKP, EK, or ALK, highlighting the need for consistent infection surveillance [53]. Yang et al. [54] identified immune rejection and postoperative inflammation as key contributors to graft failure, even in low-risk keratoconus patients. In a case report [54], Alfaraidi et al. described post-PKP infectious keratitis caused by *Elizabethkingia meningoseptica*, reinforcing the need for stringent infection control and patient education to minimize rare but severe complications.

These findings reinforce the importance of integrating both subjective and objective measures in evaluating corneal transplantation outcomes. The significant correlations we observed between VFQ-25 scores and both BCVA and contrast sensitivity emphasize the close relationship between visual function and patient-perceived quality of life. Although we did not perform a formal analysis of structural parameters (e.g., corneal topography or posterior curvature), the consistent direction of improvement across domains suggests a meaningful structure–function relationship. Future studies incorporating standardized postoperative imaging (e.g., Pentacam, SS-OCT) could further clarify these associations.

Although our focus was on keratoconus, keratoplasty also plays a transformative role in other corneal pathologies. For example, Mgbako et al. [55] reported favorable keratoplasty outcomes in patients with trachomatous corneal opacities, with graft clarity rates reaching 83% in PKP and 90% in LKP. However, their review also highlighted a lack of PROM data such as VFQ-25 in low-resource settings, indicating a need for broader implementation of standardized patient-centered assessments. [55].

Although we did not perform formal statistical correlation between tomographic variables and VFQ-25 scores, the consistent direction of improvement across both subjective and objective domains suggests a meaningful structure–function link. Future prospective studies should aim to include standardized postoperative imaging (e.g., Pentacam, SS-OCT) to allow for precise analysis of how corneal morphology restoration relates to visual function and quality of life metrics. Such integration would improve the generalizability of results and support evidence-based patient counseling.

This study has certain limitations that should be considered when interpreting the results. The sample size was relatively limited, and expanding the research across multiple centers would enhance the generalizability of the findings. Additionally, the follow-up period was restricted to one year, and longer-term evaluations could provide further insights into the durability of visual and quality of life improvements after PKP. Another limitation is the reliance on self-reported data through the VFQ-25 questionnaire, which, while validated, remains subjective. Future studies should incorporate objective measures of functional vision and include more diverse patient populations to strengthen the conclusions. Although the VFQ-25 questionnaire was adapted into Polish following standardized translation procedures, including forward and back translation and expert review, a full psychometric validation was not conducted. Cross-cultural adaptations, such as the Tamil version validated by Anand et al. [15], underscore the importance of regional linguistic and cultural sensitivity when assessing patient-reported outcomes. Future studies should aim for formal psychometric validation of translated tools to improve comparability and generalizability. This may limit the generalizability of some findings, and future studies should include formal validation of the Polish version of the questionnaire.

Furthermore, although preoperative imaging with OCT and Pentacam was performed, postoperative structural assessments were not systematically conducted in this study. This limits our ability to explore detailed structure–function relationships between anatomical restoration and perceived quality of life gains after PKP. While our correlation analysis revealed significant associations between VFQ-25 composite scores and preoperative BCVA and contrast sensitivity, incorporating standardized postoperative imaging and contrast sensitivity testing would allow for a more robust evaluation of functional and anatomical recovery. Future research should aim to include longitudinal structural assessments (e.g., anterior/posterior corneal curvature, pachymetric changes, and densitometry) alongside patient-reported outcomes to enhance generalizability, establish stronger clinical correlations, and guide personalized management in keratoconus.

## 5. Conclusions

PKP significantly improves both objective visual function and subjective quality of life in patients with advanced KC. In this study, BCVA and contrast sensitivity showed marked improvements one year after surgery, demonstrating the clinical effectiveness of PKP in restoring functional vision.

Correspondingly, VFQ-25 composite scores improved significantly, with particularly large gains observed in the domains of near and distance activities, general vision, and mental health. These results confirm that PKP not only restores visual acuity but also alleviates the psychosocial and functional burdens associated with keratoconus.

Moreover, preoperative correlation analyses revealed that subjective quality of life was more strongly associated with visual function (BCVA and contrast sensitivity) than with anatomical indicators such as corneal curvature, underscoring the importance of incorporating functional and PROMs in clinical assessment.

Altogether, our findings underscore the therapeutic value of PKP in restoring both visual function and quality of life in advanced KC. These results support the integration of PROMs into routine clinical evaluation and emphasize the need for multimodal assessment—including anatomical imaging—to guide patient-centered care.

## Figures and Tables

**Table 1:** Inclusion and exclusion criteria.

Inclusion Criteria	Exclusion Criteria
Signed informed consent to participate in the research	Refusal to participate
Age over 18 years	Age under 18 years
Confirmed diagnosis of keratoconus in the eye that underwent penetrating keratoplasty (PKP)	Absence of a confirmed keratoconus diagnosis
Polish Caucasian ethnicity	History of corneal transplantation other than PKP
	Pregnancy
	Presence of any corneal pathology other than keratoconus
	Coexisting ophthalmological conditions (e.g., glaucoma, diseases of the retina, choroid, or optic nerve)
	Presence of cardiovascular disease
	History of ocular interventions (e.g., CXL, intracorneal ring implantation, phakic IOLs)
	RGP contact lens use within two weeks prior to the preoperative examination

PKP, Penetrating Keratoplasty; CXL, Corneal Cross-Linking; IOLs, Intraocular Lenses; RGP, Rigid Gas Permeable (contact lenses).

**Table 2:** NEI VFQ-25 subscales and question types.

VFQ-25 Subscale	Type of Questions Assessed
General Health	Self-perceived overall health status
General Vision	Subjective evaluation of overall visual ability
Ocular Pain	Frequency and severity of discomfort, burning, or aching in and around the eyes
Near Vision	Difficulty with tasks requiring near focus, such as reading, cooking, or using small tools
Distance Vision	Problems with recognizing faces, reading signs, or watching television
Vision-Specific Social Functioning	Limitations in social interaction due to visual difficulties
Vision-Specific Mental Health	Emotional impact of visual impairment (e.g., frustration, worry, embarrassment)
Vision-Specific Role Difficulties	Reduced ability to fulfill work or household responsibilities due to vision
Vision-Specific Dependency	Level of reliance on others in daily functioning
Driving	Difficulties with driving in various conditions (day, night, poor weather, traffic)
Color Vision	Trouble distinguishing between different colors
Peripheral Vision	Impaired side vision or difficulty detecting objects out of the direct line of sight

**Table 3:** Vision-related quality of life scores (VFQ-25) before and after PKP in 71 patients (86 eyes).

Subscale	Before (Median [Q1–Q3], Min–Max)	After (Median [Q1–Q3], Min–Max)	Change (Median [Q1–Q3], Min–Max)	Improved (n)	Unchanged (n)	Worsened (n)	*p*-Value	Improved (%)	Unchanged (%)	Worsened (%)
General Health	50 [50–75], 0–100	75 [50–75], 0–100	0 [0–25], −50–100	4	53	29	<0.001	3.85%	61.54%	34.62%
General Vision	40 [50–60], 20–100	80 [50–80], 20–100	20 [20–40], −40–80	2	18	66	<0.001	2.56%	21.79%	75.64%
Ocular Pain	62.5 [37.5–75], 12.5–100	75 [62.5–87.5], 25–100	12.5 [0–37.5], −37.5–62.5	10	27	49	<0.001	12.82%	30.77%	56.41%
Near Activities	50 [33.33–66.67], 8.33–100	83.33 [75–100], 16.67–100	25 [14.58–50], −41.67–75	4	11	71	<0.001	5.13%	14.10%	80.77%
Distance Activities	50 [41.67–66.67], 16.67–100	91.67 [75–100], 16.67–100	25 [8.33–50], −16.66–83.33	3	17	66	<0.001	3.85%	17.95%	78.21%
Social Functioning	75 [50–87.5], 12.5–100	100 [87.5–100], 50–100	12.5 [0–37.5], −12.5–87.5	1	30	55	<0.001	1.28%	35.90%	62.82%
Mental Health	46.88 [25–68.75], 6.25–93.75	75 [57.81–87.5], 12.5–100	18.75 [0–37.5], −12.5–87.5	1	23	62	<0.001	1.28%	26.92%	71.79%
Role Difficulties	37.5 [25–50], 0–100	75 [50–87.5], 0–100	25 [0–50], −75–75	8	15	62	<0.001	10.26%	19.23%	70.51%
Dependency	75 [50–91.67], 0–100	100 [83.33–100], 0–100	12.5 [0–33.33], −66.67–91.67	4	25	56	<0.001	5.13%	29.49%	65.38%
Driving	50 [25–75], 0–100	50 [50–75], 0–100	25 [0–41.67], −91.67	1	12	38	<0.001	1.96%	23.53%	74.51%
Color Vision	100 [56.25–100], 25–100	100 [100–100], 50–100	0 [0–25], −25–75	3	50	33	<0.001	3.85%	60.26%	35.90%
Peripheral Vision	50 [50–75], 25–100	100 [75–100], 25–100	25 [0–50], −25–75	3	30	53	<0.001	3.85%	33.33%	62.82%

Data are presented as median [Q1; Q3], min–max. N refers to the number of patients. VFQ-25, Visual Function Questionnaire–25; PKP, penetrating keratoplasty.

**Table 4:** Best-corrected visual acuity (BCVA) before and after PKP in 86 eyes from 71 patients.

BCVA Before PKP	BCVA After PKP	*p*-Value (Wilcoxon Signed Rank Test)
0.05 [0.01; 0.20]	0.80 [0.60; 1.00]	<0.001

Data are presented as median [Q1; Q3]. BCVA, best-corrected visual acuity; PKP, penetrating keratoplasty.

**Table 5:** Contrast sensitivity before and after PKP in 86 eyes from 71 patients (Pelli–Robson chart).

Before PKP	After PKP	*p*-Value (Wilcoxon Signed Rank Test)
1.10 [0.90; 1.35]	1.65 [1.50; 1.80]	<0.001

Data are presented as median [Q1; Q3]. PKP, penetrating keratoplasty.

## Data Availability

The data used to support the findings of this study are included in the article. The data cannot be shared due to third-party rights and commercial confidentiality.

## Abbreviations

BCVA	Best-Corrected Visual Acuity
CXL	Corneal Cross-Linking
DALK	Deep Anterior Lamellar Keratoplasty
KC	Keratoconus
Kmean	Mean Keratometry
OCT	Optical Coherence Tomography
PKP	Penetrating Keratoplasty
PROMs	Patient-Reported Outcome Measures
QoL	Quality of Life
RGP	Rigid Gas Permeable (contact lenses)
VFQ-25	Visual Function Questionnaire—25 Items
NEI	National Eye Institute
SD	Standard Deviation
IQR	Interquartile Range
SS-OCT	Swept-Source Optical Coherence Tomography
